# Socio-spatial heterogeneity in participation in mass dog rabies vaccination campaigns, Arequipa, Peru

**DOI:** 10.1371/journal.pntd.0007600

**Published:** 2019-08-01

**Authors:** Ricardo Castillo-Neyra, Amparo M. Toledo, Claudia Arevalo-Nieto, Hannelore MacDonald, Micaela De la Puente-León, Cesar Naquira-Velarde, Valerie A. Paz-Soldan, Alison M. Buttenheim, Michael Z. Levy

**Affiliations:** 1 Department of Biostatistics, Epidemiology and Informatics, Perelman School of Medicine, University of Pennsylvania Perelman School of Medicine, Philadelphia, Pennsylvania, United States of America; 2 Zoonotic Disease Research Lab, One Health Unit, School of Public Health and Administration, Universidad Peruana Cayetano Heredia, Arequipa, Peru; 3 Department of Biology, School of Arts and Sciences, University of Pennsylvania, Philadelphia, Pennsylvania, United States of America; 4 Global Community Health and Behavioral Sciences, Tulane School of Public Health and Tropical Medicine, New Orleans, Louisiana, United States of America; 5 Department of Family and Community Health, School of Nursing, University of Pennsylvania, Philadelphia, Pennsylvania, United States of America; Centers for Disease Control and Prevention, UNITED STATES

## Abstract

To control and prevent rabies in Latin America, mass dog vaccination campaigns (MDVC) are implemented mainly through fixed-location vaccination points: owners have to bring their dogs to the vaccination points where they receive the vaccination free of charge. Dog rabies is still endemic in some Latin-American countries and high overall dog vaccination coverage and even distribution of vaccinated dogs are desired attributes of MDVC to halt rabies virus transmission. In Arequipa, Peru, we conducted a door-to-door post-campaign survey on >6,000 houses to assess the placement of vaccination points on these two attributes. We found that the odds of participating in the campaign decreased by 16% for every 100 m from the owner’s house to the nearest vaccination point (p = 0.041) after controlling for potential covariates. We found social determinants associated with participating in the MDVC: for each child under 5 in the household, the odds of participating in the MDVC decreased by 13% (p = 0.032), and for each decade less lived in the area, the odds of participating in the MDVC decreased by 8% (p<0.001), after controlling for distance and other covariates. We also found significant spatial clustering of unvaccinated dogs over 500 m from the vaccination points, which created pockets of unvaccinated dogs that may sustain rabies virus transmission. Understanding the barriers to dog owners’ participation in community-based dog-vaccination programs will be crucial to implementing effective zoonotic disease preventive activities. Spatial and social elements of urbanization play an important role in coverage of MDVC and should be considered during their planning and evaluation.

## Author summary

In Peru and other dog rabies-affected countries, mass dog vaccination campaigns (MDVC) are implemented primarily through fixed-location vaccination points: owners have to bring their dogs to the vaccination points where they receive the vaccination. To stop rabies virus transmission, a high and even dog vaccination coverage is desired. In Arequipa, Peru, following a MDVC, we conducted a door-to-door survey of >6,000 houses to assess how the placement of vaccination points affected coverage of the campaign. When comparing dog owners with similar characteristics, we found that the odds of participating in the MDVC was reduced by 16% for every 100 m distance from the nearest vaccination point. Some social conditions were also associated with participating in the MDVC: for each child under 5 in the household, odds of participating in the MDVC decreased by 13%, and for each decade less lived in the area, the odds of participating in the MDVC decreased by 8%. Distance to the vaccination point and variation in social conditions across the city play important roles in achieving coverage of MDVC and should be considered during campaign planning and evaluation.

## Introduction

The city of Arequipa is in the midst of a sustained dog rabies outbreak. The introduction of rabies virus into the city has been ascribed to the unintentional transport of rabid dogs from the rabies-endemic state of Puno during human migration [1–3], and the persistence of transmission is likely due to low coverage in the annual city-wide dog vaccination campaigns [3]. Following the detection of the outbreak in Arequipa city in 2015, the Ministry of Health of Peru (MOH) initiated additional vaccination campaigns in the city with varying intensity [4]. These additional efforts have not quelled the epidemic: more than 160 rabid dogs have been detected as of 2019.

Epidemics of dog rabies are ongoing in major urban centers across Latin America and worldwide [1,5–8]. Since bites from rabies-infected dogs cause 99% of human rabies deaths [9], the control and elimination of dog-mediated human rabies relies on a One Health strategy: mass dog rabies vaccination [8,10,11]. Dog vaccination has dramatically decreased the global burden of human rabies since 1955 [5,12,13]; in the Americas, national programs centered around mass dog vaccination have achieved enormous advances [8,14,15], reducing the incidence of dog rabies by 98% since 1983 [14]. In most rabies-affected countries, government health entities (e.g. MOH) organize annual mass dog vaccination campaigns (MDVC) that are held in outdoor settings. These campaigns are usually free of charge and voluntary [8,16] and campaign promotion varies greatly in format, content and intensity [1,17,18].

There are three non-mutually exclusive strategies used in Peru to implement MDVC: fixed vaccination posts, mobile teams setting up a temporary mobile post or conducting ‘street vaccination’, and door-to-door vaccination [19]. For the fixed-point strategy, the vaccinators wait for the dog owners to bring the dogs to a unique place. For the mobile team strategy, the vaccinators move from one location to another during the day, vaccinating dogs along their way and spending short periods (i.e. under an hour) in a location before moving on, waiting for the dog owners to bring the dogs to these moving locations. For the door-to-door strategy, vaccinators knock on doors asking to vaccinate dogs in the household. Locations of the fixed vaccination sites are typically determined by a combination of convenience and prominence of the location (e.g. the entrance to a health post, a well-known park) [11]. In Peru, routes for door-to-door and mobile team approaches may or may not be decided in advance, and teams may move during the course of the day looking for dogs along their routes.

The fixed-point strategy has been extensively used in Latin America and Africa, even though it has frequently failed to attain coverage targets [1,8,20–24]. The main reasons for its extensive application are that fixed-point vaccination is easier to implement and less costly than other strategies [18]. In many cases fixed-point is combined with other strategies, particularly when initial activities are unsuccessful [19,23,25]. However, high dog owner participation in MDVC and other dog-centered health campaigns (e.g. de-worming dogs to prevent human hydatid disease) has proven difficult to achieve in many areas [24,25]. It is necessary to understand barriers to community-based control strategies targeting dog populations in dog rabies-affected countries where coverage does not reach the minimum 70% recommended by the World Health Organization to attain herd immunity [11], much less the 80% recommended by the Pan-American Health Organization for the region [26].

**Fig 1:**
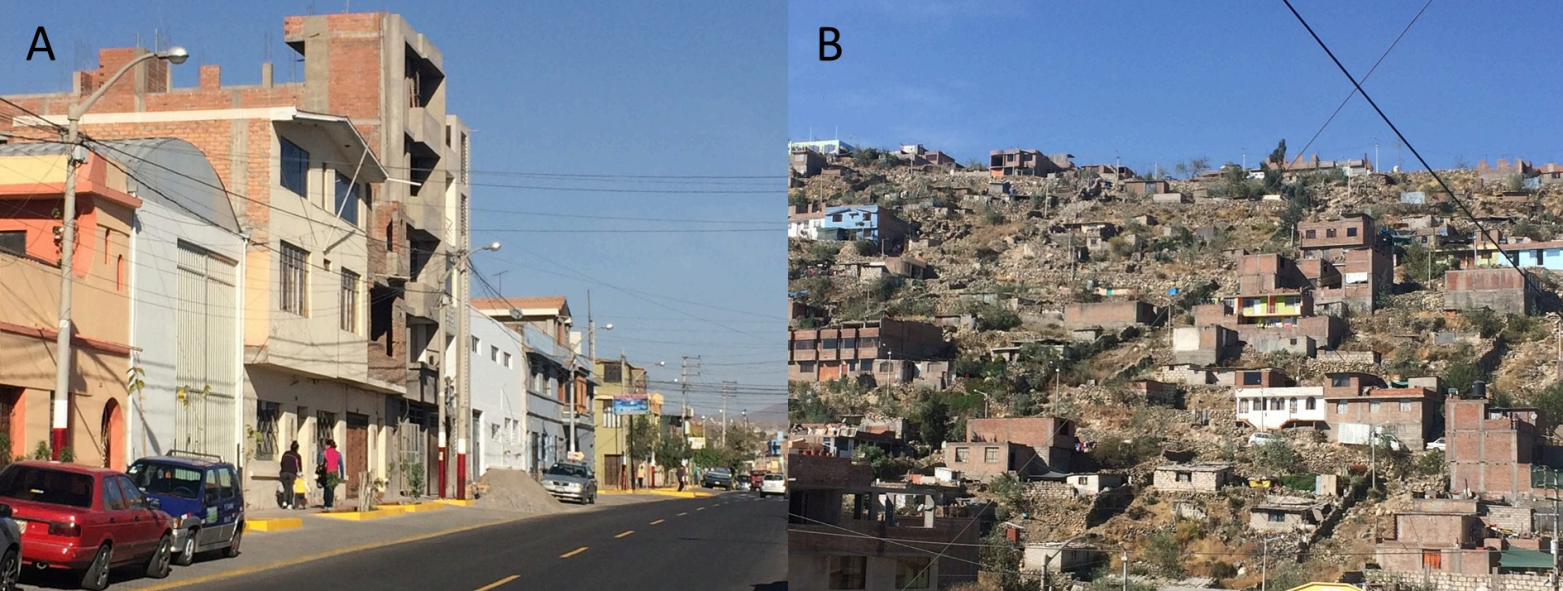
Study communities display landscape heterogeneity. A: Urban area. B: Peri-urban area.

In cities, the social and spatial aspects of urbanization can facilitate the emergence of dog rabies and complicate its control [27,28]. The city of Arequipa comprises communities spanning different stages of urbanization and different migration histories, from old established neighborhoods, to young neighborhoods, to recent invasions [29]. Within this gradient of development, young neighborhoods and recent invasions are often located on the periphery of the city (peri-urban area) and the older localities are nearer to the center (urban area) [29]. Compared to the urban area, peri-urban areas generally have lower socioeconomic status, fewer community resources, more security problems, and often more rugged and uneven terrain (Fig 1). In the city of Arequipa, the locations of rabid dogs have been associated with urban structures [27], and dog owners from areas with different levels of urbanization have reported distinct correlates of vaccinating their dogs against rabies [1]. The changing urban landscape and social processes in rapidly-growing cities have been associated with uptake of health-related services [30–33] and may be related to the low dog vaccination coverage in Arequipa. The objectives of the present study were to quantitatively assess the association between distance to a vaccination point and dog owner participation in mass dog vaccination campaigns in an urban setting, and to evaluate the effect of such distance on overall vaccination coverage and spatial distribution of participation.

## Methods

### Ethics statement

Ethical approval was obtained from Universidad Peruana Cayetano Heredia (approval number: 65369), Tulane University (approval number: 14–606720), and University of Pennsylvania (approval number: 823736). All human subjects in this study were adults.

### Study setting

The study was conducted in Alto Selva Alegre (ASA) (human population for 2015: 82,412; density: 11,902 people/km^2^), one of the 14 districts of the city of Arequipa. Arequipa, Peru’s second largest city, is home to 969,000 people and is situated at ~2,300 meters above sea level. The first detection of a rabid dog in the city of Arequipa occurred in March 2015 in ASA. By June 2016, when our data were collected, 43 rabid dogs had been detected in 8 districts, but it is assumed that number represents a small fraction of the total number of cases [27]. The 14 districts in Arequipa vary in human population size, house density, and socioeconomic status, among other variables related to canine rabies ecology. These districts are formed by contiguous neighborhoods that also vary in those characteristics, and this variation is usually associated to the level of urbanization of those neighborhoods. As new neighborhoods mature into established neighborhoods with wealthier residents, homes are improved with better quality construction material and permanent utility connections, and connectivity with the rest of the city increases with better sidewalks, roads, and transportation access. ASA spans the gradient of urban development, running from the center to the periphery, and the district continues to grow towards the outskirts of the city. In our study, participants represented either the urban or peri-urban residential areas of the city of Arequipa (Fig 1). We included 21 urban neighborhoods founded many decades ago, and 21 peri-urban neighborhoods that originated around 2000 or later.

In ASA, the MOH conducted a mass dog vaccination campaign in June 2016. The human-to-dog ratio used to estimate the dog population was 10:1 in 2015 and it was reduced to 6:1 for 2016, the year we conducted our survey. A detailed description of the mass dog vaccination campaigns can be found elsewhere [1].

### Data collection

In collaboration with the MOH, we georeferenced every stationary and mobile vaccination team during the three weekends when the campaign was implemented in ASA. Due to volunteer assistance, some dogs were vaccinated in weekdays during the campaign; we did not georeference the location of those volunteering vaccinators, mainly because their schedule was haphazard and unpredictable. We started door-to-door surveys immediately after the vaccination campaign, visiting every household in the study area and consenting and surveying only one adult (≥18) per household.

The door-to-door survey was designed based on the rabies literature and based on our qualitative studies of local communities, in which we found specific household-level barriers to vaccination [1]. We collected household variables (e.g. number of household members; number of children under 5 years old), dog owner or interviewee variables (e.g. gender; educational attainment), and dog variables (e.g. vaccination status; age). All houses in the study localities were geocoded and the survey data were linked to the household coordinates. We estimated the Euclidean distance between households and the closest vaccination point (fixed, mobile, or either).

### Statistical analysis

We estimated the total vaccination coverage in the study area and compared the coverage in urban vs. peri-urban localities with a chi-squared test. We estimated the human to dog ratio and bootstrapped it 10,000 times to estimate its confidence interval. To evaluate the baseline characteristics of households and dog owners by participation in the MDVC, we defined an ordinal outcome for houses with dogs: *no participation* (no dog vaccinated in the house), *partial participation* (some, but not all dogs in the house, vaccinated), and *full participation* in the MDVC (all dogs in the house vaccinated). We used a chi-square test to compare categorical variables with 10 or more observations per group, Fisher's exact test for categorical variables with fewer than 10 observations in any subgroup, and Mann–Whitney U test for age of the dog owner or interviewee, which did not follow a normal distribution and was truncated at 18 years. We compared the individual characteristics of vaccinated and unvaccinated dogs with chi-square for categorical variables and with Mann–Whitney U test for dog's age.

Our main objective was to assess the association between distance to the vaccination point and participation in the MDVC. For distance to the vaccination point, we used the Euclidean distance from the dog’s house to the closest vaccination point, either fixed or mobile. For participation in the MDVC we used the ordinal values described above: *no participation*, *partial participation*, and *full participation*. We compared proportional odds logistic regression (POLR), non-proportional odds logistic regression, and multinomial regression. The ordinal models were superior to the multinomial regression, and the proportional odds assumption holds for most of the covariates. Given that the categories of participation are inherently ordinal and that providing a single point estimate per covariate is more interpretable, the POLR model was favored. Based on the recent literature and our local studies [1,34,35], the following covariates were used for model building: having a dog leash at home, number of children under 5 years old at home, time living in the area, rabies status of the last place they lived in before living in the study area, number of dogs at the house, age and gender of the dog owner, and educational attainment. For rabies status we used three categories: ‘endemic’ for departments that have reported dog rabies transmission during the last decades, ‘epidemic’ for Arequipa, and ‘free of dog rabies’ for the rest of the departments. We considered transformed distance to capture non-linear effects and interactions between distance and having a leash. The fit of the alternative models to the data was compared with Akaike’s Information Criteria (AIC). We also attempted to build a hierarchical model to take into account the spatial autocorrelation within locality. However, given that within each locality there was at most one vaccination point, the variable distance from the house to the vaccination point would be unidentifiable under such hierarchical model. The final POLR model fitted with the R package MASS [36] was:

ln{Odds(Y≤k)Odds(Y>k)}=b0+b1.distance+b2.leash+b3.children+b4.recencyinthearea+b5.rabiesstatusofpreviousresidence+ε

where *k* takes the values 0 (no participation), 1 (partial participation), and 2 (full participation). All statistical tests were 2-sided, and significance level was 0.05.

We tested the spatial pattern of vaccinated and unvaccinated dogs in relation to vaccination tents for clustering using the bivariate cross K-function. This function estimates spatial dependence between two types of points (i.e. unvaccinated dogs and vaccination points) by measuring the expected number of points of type *i* within a given distance to a point of type *j* divided by the overall density of the points of type *i*. We used the Kcross function in the R package spatstat [37] to estimate deviations between the K function estimated for our data and the theoretical K function corresponding to a completely random Poisson point process for vaccinated dogs to tents and unvaccinated dogs to tents. Deviations greater than the theoretical K function indicate that the mean point count is higher than expected under complete spatial randomness (CSR) and thus some degree of clustering is present between the two event types at the indicated distance. Similarly, deviations less than the theoretical K function indicate that the mean point count is lower than expected under CSR which therefore indicates that some degree of dispersion is present between the two event types at the indicated distance.

In order to investigate the association between geolocation and the odds of canine vaccination we fitted Generalized Additive Models (GAMs) to our data using the R package MapGAM [38]. GAMs are an extension of linear regression models in which both parametric and non-parametric terms are used to estimate the outcome of interest. We used a two-dimensional locally weighted smooth (LOESS) of latitude and longitude for our non-parametric term. The LOESS smoother fits each data point by weighting it towards nearby points, where weighting is based on the distance to the point being fitted. The percentage of data points in the region that will be used to predict a particular point is referred to as the span. The optimal span size used for smoothing was determined by minimizing the AIC. We mapped the odds ratio for each point on pre-specified grids of each locality (from polygon data) and next tested the null hypothesis that the odds of each points’ vaccination status did not depend on geolocation using permutation tests. For each test, the paired latitude and longitude coordinates were randomly permuted but vaccination status was held fixed. 1000 permutations were run for each locality and contour lines encircle areas with significantly increased or decreased vaccination odds as indicated by point wise p-values computed from the permutation ranks. All models and figures were created with R [39].

## Results

Based on our survey, the estimated vaccination coverage of the MOH MDVC was only 58.1%, and it was low in both urban and peri-urban localities (58.0% vs. 58.6% respectively, chi2 = 0.086, p = 0.769). Only 3.4% of dogs were (reportedly) vaccinated in private clinics, bringing our estimated total coverage to 61.5%. Participation in our survey was higher in the urban area (88.8%) compared to the peri-urban area (61.6%) (mean = 82.0%, chi2 = 6458.5, p<0.001). The total number of dogs in the surveyed houses was 5,292 and the human to dog ratio was 3.78:1 (95% CI: 3.69:1–3.89:1), much lower than the 6:1 ratio used by the MOH for planning and evaluating the MDVC. In total, 65.3% of surveyed houses had dogs, but this number was higher in peri-urban areas (70.0% compared to 64.6% in urban areas, chi2 = 6.529, p = 0.011). For 76.9% of vaccinated dogs in the area, the person who took them to the MDVC is the person who responded to the survey. In our study area the urbanization process involves new localities being founded and settlers moving in. Accordingly, we found that the time of residency (or the year people moved into this area) was clustered at the locality level. However, we found that people living in or founding a locality do not necessarily share the same place of origin or previous residence.

When we compared the distance from each house to the closest fixed vaccination point, to the closest mobile vaccination point, and to either the closest fixed or mobile vaccination point, we found a clear gradient in distance from vaccination point from non-participant houses (farther) to houses that partially participated (closer) to houses that fully participated (closest) (Table 1). The proportion of households with children under 5 was higher in households that did not participate in the campaign (36%) compared to houses that participated fully or partially in the campaign (31% and 29% respectively; p-value = 0.019). The proportion of houses with a dog leash increases from those who did not participate, to those who participated partially, to those that fully participated in the MDVC. There were some differences in MDVC participation by migration history: people who have lived longer in ASA tend to report higher participation in the MDVC compared to those who have lived fewer years in ASA. Also, there is a slight difference in participation in the MDVC depending on rabies status of previous residence, with more people participating in the campaign, partially or fully, if they were migrants from a rabies endemic area (Table 1). Other variables, such as educational attainment, the proportion of female dog owners or interviewees, and the proportion of households in urban localities were all similar in those households that participated fully or partially in the campaign compared to those households that did not participate of the campaign (Table 1). Having multiple dogs is a prerequisite to be in the partial participation group; therefore, houses with partial participation had on average more dogs, but the number of dogs per house was very similar in houses that participated fully compared to those that did not participate in the campaign (Table 1). Those who did not participate in the MDVC reported more frequently not knowing about the campaign before it happened (Table 1), but many of them reported learning about the campaign the same day it occurred. In S1 Table, we report the media channels through which they learned about the campaign either before it occurred or the same day it occurred.

**Table 1:** Characteristics of the dog owner population in the study area by participation in the MDVC, Arequipa City, Peru, 2016.

Interviewee/Owner Variables	Vaccinatedno dog(n = 1,017)	Vaccinatedsome dogs(n = 176)	Vaccinatedall dogs(n = 1,434)	p
Distance to any closest vaccination point–Mean (SD)	125m (96)	117m (114)	103m (83)	<0.001 ^a^
Distance to closest fixed vaccination point–Mean (SD)	501m (479)	490m (420)	461m (422)	0.119 ^a^
Distance to closest mobile vaccination point–Mean (SD)	137m (101)	127m (119)	114m (93)	<0.001 ^a^
Number of dogs in houses with dogs–Mean (SD)	1.79 (1.10)	2.96 (1.24)	1.80 (1.05)	<0.001 ^a^
Houses with children under 5	36.4%	28.9%	30.8%	0.019 ^b^
Age of dog owner/interviewee–Median (IQR)	39 years(28–52)	40 years(29–50)	42 years(30–53)	0.024 ^c^
Time living in ASA if migrant–Mean (SD)	18.1 years(13.7)	21.2 years(13.9)	22.1 years(14.8)	<0.001 ^a^
Area before living in ASA				0.036 ^b^
Rabies epidemic (Arequipa)	90.7%	94.1%	89.8%	
Rabies endemic (Puno)	1.6%	2.4%	3.3%	
Rabies free (rest of country)	7.7%	3.6%	6.9%	
Educational attainment				0.509 ^b^
Illiterate	0.6%	0.5%	0.6%	
Primary school	11.5%	11.4%	13.6%	
High school	43.2%	48.3%	41.1%	
Technical academy	21.2%	15.3%	20.6%	
University	22.3%	23.3%	23.3%	
Preferred not to respond	1.2%	1.1%	0.7%	
Owns at least one dog leash	51.0%	54.5%	60.1%	<0.001 ^b^
Female interviewees/owners	64.6%	73.3%	64.4%	0.032 ^b^
Did not know about the campaign before it happened	15.4%	2.3%	1.3%	<0.001 ^b^
Live in urban locality (vs. peri-urban)	78.8%	78.1%	79.4%	0.002 ^b^

*p* values estimated with

^a^ one-way ANOVA

^b^ Chi square test; and

^c^ Kruskal Wallis test.

Compared to vaccinated dogs, unvaccinated dogs were older, were more likely female, had more free access to the street, had owners with no leash for them at home, and were less likely to be walked. Multipurpose dogs (dogs reported as guard and company dogs) were more likely to be vaccinated (Table 2). Based on the stated source of the dog, those received as gifts are more likely to be vaccinated and dogs born at home or adopted/picked on the street are less likely to be vaccinated (Table 2). Being considered purebred or being spayed/neutered was not associated with dog vaccination status (Table 2).

**Table 2:** Dog characteristics by vaccination status in the MDVC (dogs vaccinated privately not included).

Dog Variables	Unvaccinated dog (n = 2,019)	Vaccinated dog (n = 2,800)	p
Age of dogs–Mean (SD)	24 months (12–60)	20 months (10–54)	0.039 ^a^
Female dogs	42.1%	36.8%	<0.001 ^b^
Free access to the street	20.6%	25.7%	0.002 ^b^
Leash for the dog	33.8%	45.3%	<0.001 ^b^
Dogs that are walked, either with or without leash.	55.9%	66.9%	<0.001 ^b^
Dog function			<0.001 ^b^
Company	52.0%	49.0%	
Protection	23.4%	20.9%	
Company & Protection	24.6%	29.6%	
Breeder/business	0.0%	0.5%	
Dog source			<0.001 ^b^
Gift	51.2%	56.5%	
Bought at market/store	17.4%	18.3%	
Born at home	15.6%	12.3%	
Adopted/Picked on the street	12.3%	10.8%	
Bought from friend/neighbor	2.6%	2.0%	
Do not know	1.0%	0.0%	
Purebred as reported by interviewee	25.7%	24.9%	0.589 ^b^
Spayed/Neutered	3.5%	2.6%	0.157 ^b^

*p* values estimated with

^a^ Student’s t-test; and

^b^ Chi square test.

In the multivariable regression analysis, we found that distance to the vaccination site is strongly associated with participation of the MDVC. The odds of participating in the MDVC were 16% lower for someone who lived 100 meters farther from the vaccination point after adjusting for other covariates and this difference was statistically significant (Table 3). Those having a dog leash at home had 35% higher odds of participation in the MDVC, either fully or partially, compared to those who did not have a dog leash after adjusting for other covariates. The odds of participating in the MDVC, either fully or partially, were 13% lower for each additional child under 5 years at home, after adjusting for other covariates. Migration history was associated with participating in the MDVC; participation was lower in those who migrated more recently to the study area (8% lower odds of participation for each decade less lived in the area, after adjusting for other covariates). Another component of migration history, the previous residence region, was also associated with participating in the MDVC: those whose previous residence was a rabies-free region or was any district within Arequipa were 23% to 32% less likely to participate in the campaign compared to those whose previous residence was a rabies-endemic region, after adjusting for other covariates (Table 3). Demographic variables such as owner’s/interviewee’s age, gender and educational attainment, and other household-level variables, such as number of dogs at the house, were dropped during model selection because they neither improved the fit of the model nor were statistically associated with participating in the MDVC.

**Table 3:** Factors associated with participating in the Mass Dog Vaccination Campaign, Arequipa City, Peru, 2016.

Variable	OR	95% CI	p
Distance to closest vaccination point (100m)	0.84	0.76–0.98	0.041
Having a leash at home	1.35	1.10–1.61	0.003
Number of children U5Y at home	0.87	0.78–0.97	0.032
Recency in ASA (1 fewer decade)	0.92	0.90–0.94	<0.001
Rabies status of previous residence			
Endemic for rabies	Ref.		
Epidemic for rabies	0.77	0.63–0.93	0.006
Free of rabies	0.68	0.57–0.82	<0.001

Odds ratios estimated with multiple proportional odds logistic regressions.

**Fig 2:**
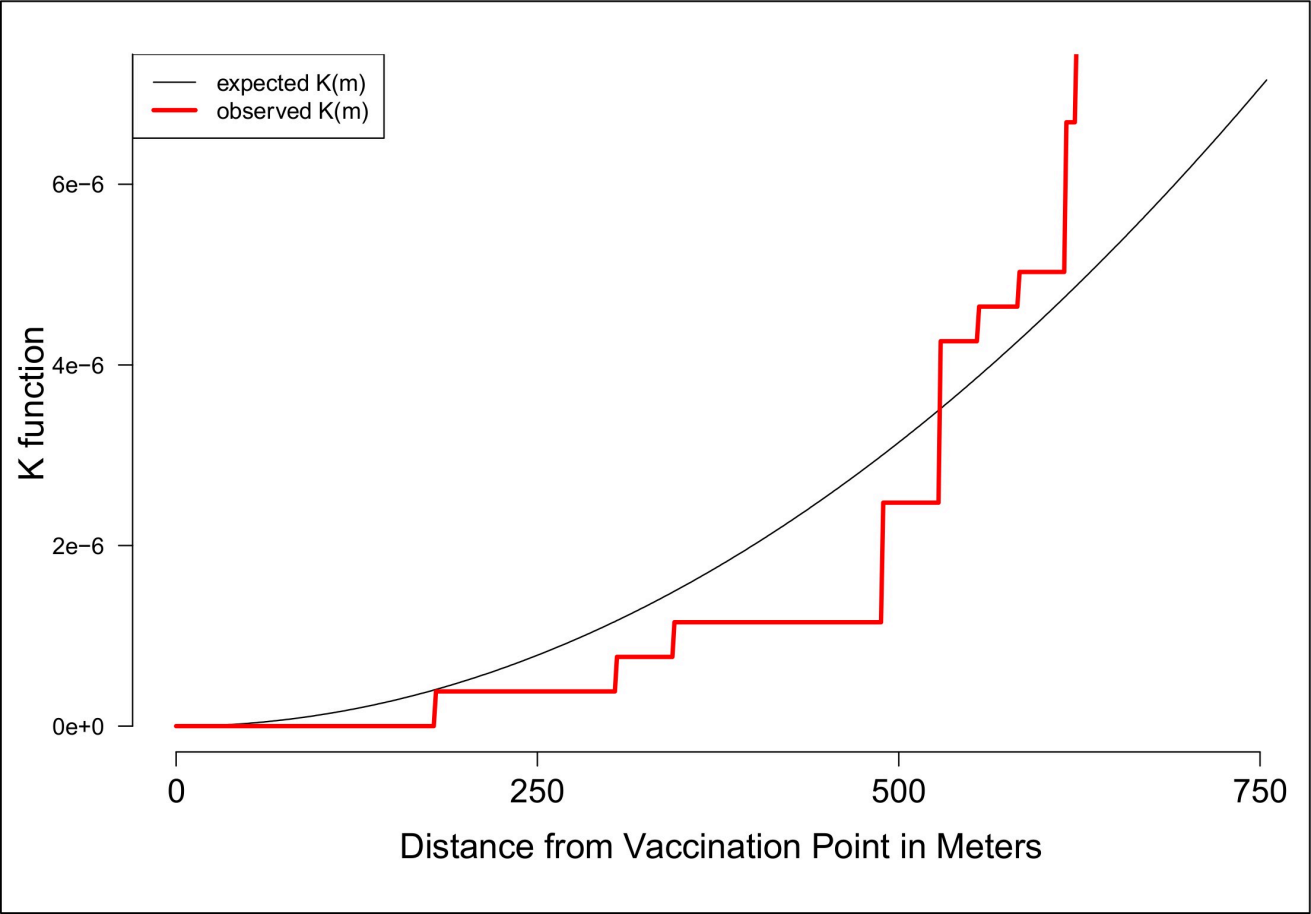
Clustering of unvaccinated dogs as a function of distance from the dog owner's house to the closest vaccination point.

**Fig 3:**
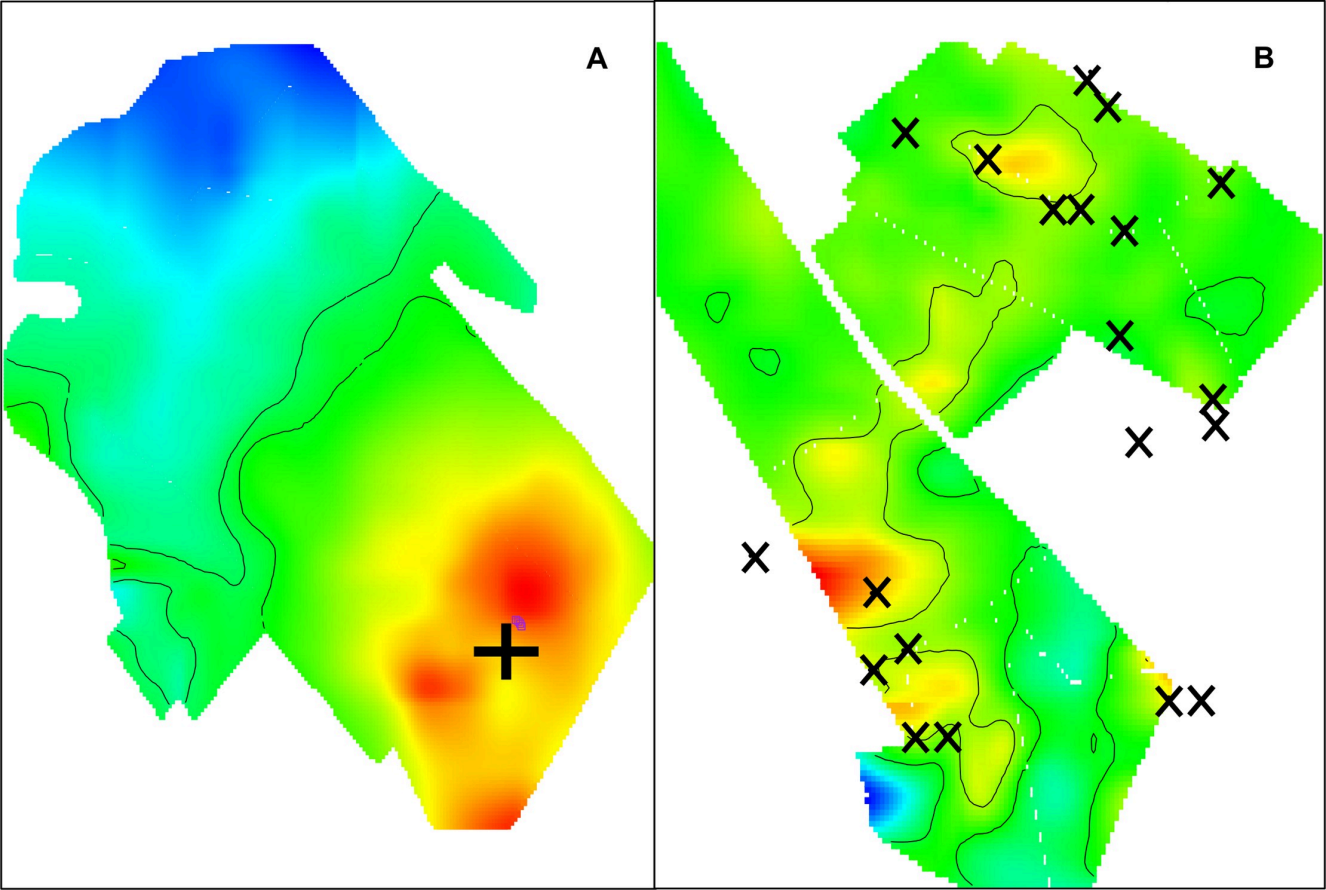
Spatial odds ratios for participating in the MDVC in a locality served by a fixed vaccination point (cross in A), and by a mobile team (in B, X’s represent locations where the mobile team stopped to wait for dogs or to vaccinate dogs). Created with MapGAM package [38] in R [39].

The significant association between distance to the vaccination point and odds of participating in the MDVC has consequences for the distribution of unvaccinated dogs in the area. We observed spatial clustering of unvaccinated owned dogs as a function of the distance from the house to the vaccination point. These pockets of unvaccinated dogs closer to each other than expected by chance occur at 500 meters from the vaccination point or further (Fig 2). We also analyzed the spatial odds of participating in the MDVC, that is, the association between their specific geolocation and the vaccination point. For areas served by fixed-point vaccination, there was a clear smooth spatial effect with higher odds of participating for houses closer to the vaccination point and a decreasing gradient farther away from the vaccination point. The spatial effect of the fixed-point vaccination strategy creates two clearly defined zones: a large zone with statistically significantly high odds of participation in the MDVC and another large zone with statistically significantly low odds of participation in the MDVC (Fig 3). For areas served by mobile teams, there were more spots of significant low and high odds of participation in the MDVC and these spots were spread in the study area without a clear association between the spots and the locations where the vaccination teams stopped to wait for dogs or to vaccinate dogs (Fig 3).

## Discussion

In Arequipa Peru, the social and spatial aspects of urbanization facilitate the emergence of dog rabies and complicate its control. In 21 urban and 21 peri-urban localities in Arequipa, Peru, we found low vaccination coverage and coverage that was spatially uneven. We found a strong effect of a potential proximal determinant of participation in the MDVC: distance to the vaccination point. The unadjusted data show a clear negative gradient with higher levels of participation in the MDVC at shorter distances to the vaccination point. After accounting for other important individual- and household-level variables, distance to the vaccination point remains an important factor associated with participating in the campaign. The association between distance to and participation in the MDVC also impacts the spatial distribution of vaccination. We found areas with statistically significant lower odds of dogs being vaccinated, and the LOESS smoother maps correlated well with maps of vaccination coverage. Therefore, it seems that both fixed point and mobile team canine vaccination approaches produced spatially heterogeneous vaccination coverage. However, we found that vaccination coverage was more “patchy” in localities served by mobile vaccination teams. This combination of mobile and fixed points was used also in 2015, but the same localities are not always served with the same approach (e.g. a locality that was served with mobile teams in 2015 could be served with fixed point vaccination in 2016). As others have reported [40], there is potential that in some localities owners in 2016 expected that the MDVC would be brought to their doors and did not plan or intend to bring the dogs to the fixed points in their areas. Spatially heterogeneous vaccination coverage is undesirable for dog rabies control and elimination. Townsend et al. [41] found that such patchy coverage can "profoundly damage prospects of elimination […] by creating pockets where rabies could persist", and modeled patchy coverage within 1 km^2^ cell grids. The low coverage ‘patches’ in our study had smaller areas than 1 km^2^, thus the potential for a threat to elimination efforts may be different or non-existent. However, it is unknown if these ‘patches’ are large enough to sustain rabies transmission in these densely populated areas or if there are much larger ‘patches’ in other parts of the city out of our study areas.

Many studies have explored logistical, informational, social and structural barriers for dog rabies vaccination experienced by owners in rabies-affected areas [1,17,18,23,24,40,42–52]. Two of the most common reasons identified are difficulty handling the dogs [1,23,24,48,49,51,52] and lack of time [1,24,48,49,51,52]. These two logistical barriers are correlated with a less studied element: the distance to the vaccination campaigns. Distance to health services has been fairly well studied in terms of availability of and access to health care and impact on health, especially for maternal health, treatment and prevention of chronic diseases and treatment adherence for infectious diseases [53–55], but not as much for preventing infectious diseases. Some rural studies mention distance as a potential factor for low dog vaccination coverage [23,56] and two studies directly evaluated the association between distance and overall rural villages vaccination coverage [49] and the association between distance and attendance at the MDVC in Sub-Saharan Africa [18]. In the sub-Saharan Africa study, researchers found that distance in dispersed communities have an impact on MDVC attendance [18]. Given the hilly landscape with rare direct paths between houses and vaccination points, they estimated the shortest-path distance for their analysis to take into consideration the long and tortuous routes dog owners had to follow to visit the vaccination sites. In our study area, with non-dispersed urbanized highly-populated localities and high density of street intersections that increase walkability, distance is still an important proximal explanation for low participation in the vaccination campaigns.

Our study area consisted of urban and peri-urban localities. Surprisingly, there were no clear differences in participation in the MDVC between these two groups. However, the distribution of other proximal rabies-related characteristics is different between them (e.g. more free-roaming dogs in peri-urban areas, more neutered/spayed dogs in urban areas). There are other social determinants that provide more distal explanations for participation in the MDVC. In previous focus groups conducted by our team, young females reported that having a baby at home could prevent them from participating in the campaign [1]. Similarly, we found that households with children under 5 years old were less likely to vaccinate their dogs compared to houses without children, and each additional child under 5 reduced the odds of vaccinating the dogs in the house. This insight suggests an opportunity to increase participation in the MDCV by framing the decision to vaccinate as an action taken to protect children in the household from rabies. There was also a clear difference in participation between those who lived in a dog rabies-endemic area before living in Arequipa. A possible explanation for that difference is higher awareness among that group. In our focus groups, we found low awareness and low perception of severity among residents of Arequipa [1]. Interestingly, another component of migration history was also associated with participation in the MDVC: time living in the area. This phenomenon has been observed for the utilization of other health services in different settings and populations [53–55]. Migration, settlement and adaptation are processes that take time and are necessary for the uptake of health services [56–59], and could be influencing the participation in the MDVC. Importantly, in the peri-urban localities there are more households with children under 5, more recent migrants, and more people whose previous residence was in a rabies-affected region.

Our study has a number of limitations. Other studies have focused attention on dog-level variables (e.g. sex, age, function) that might be associated to participation in MDVC [18]. We did not analyze these; rather we focus on owner and community characteristics that can be utilized by the health authorities (who rarely have the opportunity to collect detailed house-by-house information) to increase participation in MDVC. Vaccination status and access to the street were reported by the owner/interviewee and we did not request proof of vaccination (e.g. vaccination certificate). Given the bad publicity in the local media about owned free roaming unvaccinated dogs and the authorities’ threats to fine ‘irresponsible’ dog owners, there is potential for social desirability bias to inflate our estimate of vaccination coverage and deflate that of the proportion of dogs that have access to the street. We did not ask the interviewees to show the vaccination certificate they receive at the MDVC since many of them do not save these certificates. We used Euclidean distances, which are only a proxy for the real distance traveled by individuals to a vaccination post, and we did not include terrain elevation or slope which might affect walkability and the effect of distance on participation in the MDVC. Additionally, it is important to note that the MDVC in Peru only targets owned dogs. Our objective was to evaluate barriers to participation in the MDVC, so we only estimated the vaccination coverage in the owned dog population. The MDVC ignores important subpopulations for which size estimates are lacking: community dogs, strays, and feral dogs. The minimum vaccination coverage established by WHO (70%) first with empirical data [57,58] and afterwards validated with rigorous modeling [59] was directed to dogs at risk of rabies so the low coverage we found must be even lower when unowned dogs are considered.

Dog rabies virus transmission continues in Arequipa, putting at risk millions of people in the city and the surrounding departments. Dog-focused public health strategies are not limited to rabies: deworming dogs to prevent human echinococcosis [60], use of insecticide treatment [61] or vaccines [62] on dogs to prevent human Leishmaniasis or Chagas disease, are just a few examples. These programs, if they are to be successful, require high coverage and even spatial distribution in their implementation. The same approaches to reach the appropriate levels of community participation that might have worked in the 1980s are not working today. Understanding the barriers for dog owners’ participation in community-based programs will be crucial to implement effective zoonotic disease preventive activities. Distance to health services and the heterogeneous social composition of growing cities have to be examined when designing field programs to protect against zoonotic diseases.

## Supporting information

S1 Alternative Language Abstract Resumen en español. (DOCX)

S1 Table Reported exposure to MDVC communication channels by participation level. (DOCX)

## References

[1] Castillo-Neyra R, Brown J, Borrini K, Arevalo C, Levy MZ, Buttenheim A, et al Barriers to dog rabies vaccination during an urban rabies outbreak: Qualitative findings from Arequipa, Peru. Recuenco S, editor. PLoS Negl Trop Dis. 2017;11: e0005460 10.1371/journal.pntd.0005460 28306717 PMC5371379

[2] Castillo-Neyra R, Levy MZ, Náquira C. [Effect of free-roaming dogs culling on the control of canine rabies]. Rev Peru Med Exp Salud Publica. 2016;33: 772–779. 10.17843/rpmesp.2016.334.2564 28327849

[3] Ministerio de Salud del Perú—Dirección General de Epidemiología. Alerta Epidemiológica [Epidemiologic Alert]. Lima; 2015. Report No.: AE-DEVE N. 003–2015.

[4] El Presidente de la República. Decreto Supremo que declara en Emergencia Sanitaria por el plazo de noventa (90) días calendario, a la provincia de Arequipa del departamento de Arequipa [Internet]. 4 2, 2016 pp. 582224–582229. Available: busquedas.elperuano.pe/normaslegales/decreto-supremo-que-declara-en-emergencia-sanitaria-por-el-p-decreto-supremo-n-015-2016-sa-1363166-5/

[5] Aréchiga Ceballos N, Karunaratna D, Aguilar Setién A. Control of canine rabies in developing countries: key features and animal welfare implications. Rev—Off Int Epizoot. 2014;33: 311–321. 25000804 10.20506/rst.33.1.2278

[6] Wallace RM, Reses H, Franka R, Dilius P, Fenelon N, Orciari L, et al Establishment of a Canine Rabies Burden in Haiti through the Implementation of a Novel Surveillance Program. PLoS Negl Trop Dis. 2015;9: e0004245 10.1371/journal.pntd.0004245 26600437 PMC4657989

[7] Widdowson MA, Morales GJ, Chaves S, McGrane J. Epidemiology of urban canine rabies, Santa Cruz, Bolivia, 1972–1997. Emerging Infect Dis. 2002;8: 458–461. 10.3201/eid0805.010302 11996678 PMC2732486

[8] Vigilato MAN, Clavijo A, Knobl T, Silva HMT, Cosivi O, Schneider MC, et al Progress towards eliminating canine rabies: policies and perspectives from Latin America and the Caribbean. Philos Trans R Soc Lond, B, Biol Sci. 2013;368: 20120143 10.1098/rstb.2012.0143 23798691 PMC3720041

[9] Fooks AR, Banyard AC, Horton DL, Johnson N, McElhinney LM, Jackson AC. Current status of rabies and prospects for elimination. The Lancet. 2014;384: 1389–1399. 10.1016/S0140-6736(13)62707-5 PMC7159301 24828901

[10] Hampson K, Coudeville L, Lembo T, Sambo M, Kieffer A, Attlan M, et al Estimating the global burden of endemic canine rabies. PLoS Negl Trop Dis. 2015;9: e0003709 10.1371/journal.pntd.0003709 25881058 PMC4400070

[11] World Health Organization. WHO Expert Consultation on Rabies. Second report. World Health Organ Tech Rep Ser. 2013;: 1–139. 24069724

[12] Franka R, Smith TG, Dyer JL, Wu X, Niezgoda M, Rupprecht CE. Current and future tools for global canine rabies elimination. Antiviral Res. 2013;100: 220–225. 10.1016/j.antiviral.2013.07.004 23871797

[13] Lackay SN, Kuang Y, Fu ZF. Rabies in small animals. The Veterinary clinics of North America Small animal practice. 2008;38: 851–859. 10.1016/j.cvsm.2008.03.003 18501283 PMC2518964

[14] Clavijo A, Del Rio Vilas VJ, Mayen FL, Yadon ZE, Beloto AJ, Vigilato MAN, et al Gains and future road map for the elimination of dog-transmitted rabies in the Americas. Am J Trop Med Hyg. The American Society of Tropical Medicine and Hygiene; 2013;89: 1040–1042. 10.4269/ajtmh.13-0229 24306030 PMC3854880

[15] Vigilato MAN, Cosivi O, Knobl T, Clavijo A, Silva HMT. Rabies Update for Latin America and the Caribbean. Emerging Infect Dis. 2013;19: 678–679. 10.3201/eid1904.121482 23750499 PMC3647423

[16] Davlin SL, Vonville HM. Canine rabies vaccination and domestic dog population characteristics in the developing world: a systematic review. Vaccine. 2012;30: 3492–3502. 10.1016/j.vaccine.2012.03.069 22480924

[17] Cleaton JM, Wallace RM, Crowdis K, Gibson A, Monroe B, Ludder F, et al Impact of community-delivered SMS alerts on dog-owner participation during a mass rabies vaccination campaign, Haiti 2017. Vaccine. 2018;36: 2321–2325. 10.1016/j.vaccine.2018.03.017 29580642 PMC6066789

[18] Mazeri S, Gibson AD, Meunier N, Bronsvoort BMD, Handel IG, Mellanby RJ, et al Barriers of attendance to dog rabies static point vaccination clinics in Blantyre, Malawi. Rodriguez-Barraquer I, editor. PLoS Negl Trop Dis. 2018;12: e0006159 10.1371/journal.pntd.0006159 29324737 PMC5783422

[19] World Health Organization. WHO Expert Consultation on Rabies. World Health Organization technical report series Geneva; 2018 pp. 1–139. 24069724

[20] Cleaveland S, Kaare M, Tiringa P, Mlengeya T, Barrat J. A dog rabies vaccination campaign in rural Africa: impact on the incidence of dog rabies and human dog-bite injuries. Vaccine. 2003;21: 1965–1973. 10.1016/s0264-410x(02)00778-8 12706685

[21] Jibat T, Hogeveen H, Mourits MCM. Review on dog rabies vaccination coverage in Africa: a question of dog accessibility or cost recovery? Rupprecht CE, editor. PLoS Negl Trop Dis. 2015;9: e0003447 10.1371/journal.pntd.0003447 25646774 PMC4315526

[22] Gsell AS, Knobel DL, Kazwala RR, Vounatsou P, Zinsstag J. Domestic dog demographic structure and dynamics relevant to rabies control planning in urban areas in Africa: the case of Iringa, Tanzania. BMC Vet Res. 2012;8: 236 10.1186/1746-6148-8-236 23217194 PMC3534358

[23] Kaare M, Lembo T, Hampson K, Ernest E, Estes A, Mentzel C, et al Rabies control in rural Africa: Evaluating strategies for effective domestic dog vaccination. Vaccine. 2009;27: 152–160. 10.1016/j.vaccine.2008.09.054 18848595 PMC3272409

[24] Muthiani Y, Traoré A, Mauti S, Zinsstag J, Hattendorf J. Low coverage of central point vaccination against dog rabies in Bamako, Mali. Prev Vet Med. 2015;120: 203–209. 10.1016/j.prevetmed.2015.04.007 25953653

[25] Perry BD, Kyendo TM, Mbugua SW, Price IE, Varma S. Increasing rabies vaccination coverage in urban dog populations of high human population density suburbs: a case study in Nairobi, Kenya. Prev Vet Med. 1995;22: 137–142. 10.1016/0167-5877(94)00407-A

[26] Schneider MC, Belotto A, Adé MP, Hendrickx S, Leanes LF, Rodrigues MJ de F, et al Current status of human rabies transmitted by dogs in Latin America. Cad Saude Publica. 2007;23: 2049–2063. 17700940 10.1590/s0102-311x2007000900013

[27] Castillo-Neyra R, Zegarra E, Monroy Y, Bernedo R, Cornejo-Rosello I, Paz-Soldan V, et al Spatial Association of Canine Rabies Outbreak and Ecological Urban Corridors, Arequipa, Peru. Tropical Medicine and Infectious Disease. 2017;2: 38 10.3390/tropicalmed2030038 30270895 PMC6082090

[28] Burgos-Cáceres S. Canine Rabies: A Looming Threat to Public Health. Animals (Basel). 2011;1: 326–342. 10.3390/ani1040326 26486619 PMC4513477

[29] Levy MZ, Barbu CM, Castillo-Neyra R, Quispe Machaca VR, Ancca Juárez J, Escalante-Mejia P, et al Urbanization, land tenure security and vector-borne Chagas disease. Proc Biol Sci. 2014;281: 20141003 10.1098/rspb.2014.1003 24990681 PMC4100517

[30] Zhang L, Li X, Yang H, Mao R, Zhao Q. Health status and health-seeking behaviour between interprovincial and intraprovincial rural-to-urban young migrants in Nanjing China. Asia-Pacific Population Journal. 2011;26: 39–54.

[31] Li Y. Understanding health constraints among rural-to-urban migrants in China. Qual Health Res. 2013;23: 1459–1469. 10.1177/1049732313507500 24122513

[32] Benyoussef A, Cutler JL, Levine A, Mansourian P, Phan-Tan T, Baylet R, et al Health effects of rural-urban migration in developing countries—Senegal. Soc Sci Med. 1974;8: 243–262. 4852227 10.1016/0037-7856(74)90093-6

[33] Carrillo-Larco RM, Bernabé-Ortiz A, Pillay TD, Gilman RH, Sanchez JF, Poterico JA, et al Obesity risk in rural, urban and rural-to-urban migrants: prospective results of the PERU MIGRANT study. Int J Obes (Lond). 2015 10.1038/ijo.2015.140 26228458 PMC4677453

[34] Taylor LH, Wallace RM, Balaram D, Lindenmayer JM, Eckery DC, Mutonono-Watkiss B, et al The Role of Dog Population Management in Rabies Elimination-A Review of Current Approaches and Future Opportunities. Front Vet Sci. Frontiers; 2017; 4: 109 10.3389/fvets.2017.00109 28740850 PMC5502273

[35] Sambo M, Lembo T, Cleaveland S, Ferguson HM, Sikana L, Simon C, et al Knowledge, Attitudes and Practices (KAP) about Rabies Prevention and Control: A Community Survey in Tanzania. Rupprecht CE, editor. PLoS Negl Trop Dis. 2014;8: e3310 10.1371/journal.pntd.0003310 25473834 PMC4256472

[36] Venables WN, Ripley BD. Modern Applied Statistics with S. 4 ed New York: Springer; 2002.

[37] Baddeley A, Turner R. spatstat: An RPackage for Analyzing Spatial Point Patterns. Journal of Statistical Software. 2005;12 10.18637/jss.v012.i06

[38] Bai L, Bartell S, Bliss R, Vieira V. MapGAM: Mapping Smoothed Effect Estimates from Individual-Level Data [Internet]. 1st ed. Available: https://CRAN.R-project.org/package=MapGAM

[39] Team. R: A Language and Environment for Statistical Computing [Internet]. Vienna, Austria: R Foundation for Statistical Computing; 2013 pp. 1–3604. Available: http://www.R-project.org/

[40] Durr S, Mindekem R, Kaninga Y, Doumagoum Moto D, Meltzer MI, Vounatsou P, et al Effectiveness of dog rabies vaccination programmes: comparison of owner-charged and free vaccination campaigns. Epidemiol Infect. 2009;137: 1558–1567. 10.1017/S0950268809002386 19327197

[41] Townsend SE, Sumantra IP, Pudjiatmoko, Bagus GN, Brum E, Cleaveland S, et al Designing programs for eliminating canine rabies from islands: Bali, Indonesia as a case study. Rupprecht CE, editor. PLoS Negl Trop Dis. 2013;7: e2372 10.1371/journal.pntd.0002372 23991233 PMC3749988

[42] Widyastuti MDW, Bardosh KL, Sunandar, Basri C, Basuno E, Jatikusumah A, et al On dogs, people, and a rabies epidemic: results from a sociocultural study in Bali, Indonesia. Infectious Diseases of Poverty. 2015;4: 60 10.1186/s40249-015-0092-7 26137295 PMC4486702

[43] Bardosh K, Sambo M, Sikana L, Hampson K, Welburn SC. Eliminating rabies in Tanzania? Local understandings and responses to mass dog vaccination in Kilombero and Ulanga districts. Rupprecht CE, editor. PLoS Negl Trop Dis. 2014;8: e2935 10.1371/journal.pntd.0002935 24945697 PMC4063706

[44] Serebe SG, Tadesse KA. Study on community knowledge, attitude and practice of rabies in and nearby Gondar town, North West Ethiopia. Journal of Public and Epidemiology. 2014; 6(12): 429–435.

[45] Kabeta T, Deresa B, Tigre W, Ward MP, Mor SM. Knowledge, Attitudes and Practices of Animal Bite Victims Attending an Anti-rabies Health Center in Jimma Town, Ethiopia. Zinsstag J, editor. PLoS Negl Trop Dis. 2015;9: e0003867 10.1371/journal.pntd.0003867 26114573 PMC4482645

[46] De Ramos M, Bravo LC. Knowledge, attitudes, and practices of community regarding animals bite and rabies. Paediatric Infectious Disease Society of the Philippines. 2004 8(1): 24–32.

[47] Singh US, Choudhary SK. Knowledge, attitude, behavior and practice study on dog-bites and its management in the context of prevention of rabies in a rural community of Gujarat. Indian Journal of Community Medicine. 2005 30(3): 81–83.

[48] Kayali U, Mindekem R, Yemadji N. Coverage of pilot parenteral vaccination campaign against canine rabies in N'Djamena, Chad. Bulletin of the World Health Organization. 2003 81(10): 739–44. 14758434 PMC2572337

[49] Minyoo AB, Steinmetz M, Czupryna A. Incentives Increase Participation in Mass Dog Rabies Vaccination Clinics and Methods of Coverage Estimation Are Assessed to Be Accurate. PLoS Negl Trop Dis 2015 9(12):e0004221 10.1371/journal.pntd.0004221 26633821 PMC4669116

[50] Lembo T, Hampson K, Haydon DT, Craft M, Dobson A, Dushoff J, et al Exploring reservoir dynamics: a case study of rabies in the Serengeti ecosystem. The Journal of Applied Ecology. 2008;45: 1246–1257. 10.1111/j.1365-2664.2008.01468.x 22427710 PMC3303133

[51] Lapiz SMD, Miranda MEG, Garcia RG, Daguro LI, Paman MD, Madrinan FP, et al Implementation of an intersectoral program to eliminate human and canine rabies: the Bohol Rabies Prevention and Elimination Project. Zinsstag J, editor. PLoS Negl Trop Dis. 2012;6: e1891 10.1371/journal.pntd.0001891 23236525 PMC3516573

[52] Ferguson EA, Hampson K, Cleaveland S, Consunji R, Deray R, Friar J, et al Heterogeneity in the spread and control of infectious disease: consequences for the elimination of canine rabies. Sci Rep. 2015;5: 18232 10.1038/srep18232 26667267 PMC4678884

[53] Jacobs B, Ir P, Bigdeli M, Annear PL, Van Damme W. Addressing access barriers to health services: an analytical framework for selecting appropriate interventions in low-income Asian countries. Health Policy and Planning. 2012;27: 288–300. 10.1093/heapol/czr038 21565939

[54] Bissonnette L, Wilson K, Bell S, Shah TI. Neighbourhoods and potential access to health care: The role of spatial and aspatial factors. Health & Place. 2012;18: 841–853. 10.1016/j.healthplace.2012.03.007 22503565

[55] Hiscock R, Pearce J, Blakely T, Witten K. Is Neighborhood Access to Health Care Provision Associated with Individual-Level Utilization and Satisfaction? Health Services Research. 2008;43: 2183–2200. 10.1111/j.1475-6773.2008.00877.x 18671752 PMC2613990

[56] Cleaveland S. The epidemiology of rabies and canine distemper in the Serengeti, Tanzania. 10.17037/PUBS.00682291

[57] Beran GW. Urban Rabies In: Baer GM, editor. The Natural History of Rabies. 2nd ed Boca Raton; 1991 pp. 427–443.

[58] World Health Organization. Guidelines for Dog Rabies Control. Geneva: WHO; 1987. Report No.: VPH/83.43 Rev. 1.

[59] Coleman PG, Dye C. Immunization coverage required to prevent outbreaks of dog rabies. Vaccine. 1996;14: 185–186. 10.1016/0264-410x(95)00197-9 8920697

[60] Fu Q, Han X-M, Wang L-Y, Zhaxi S-M, Ma X, Wang Y-S, et al Effect of control pattern with emphasis on canine deworming in a pilot of echinococcosis in highly endemic area, southern Qinghai Plateau. Zhongguo Ji Sheng Chong Xue Yu Ji Sheng Chong Bing Za Zhi. 2011;29: 293–295. 21972606

[61] Loza A, Talaga A, Herbas G, Canaviri RJ, Cahuasiri T, Luck L, et al Systemic insecticide treatment of the canine reservoir of Trypanosoma cruzi induces high levels of lethality in Triatoma infestans, a principal vector of Chagas disease. Parasites & Vectors. BioMed Central; 2017;10: 344 10.1186/s13071-017-2278-2 28724448 PMC5518140

[62] Aparicio-Burgos JE, Zepeda-Escobar JA, de Oca-Jiménez RM, Estrada-Franco JG, Barbabosa-Pliego A, Ochoa-García L, et al Immune protection against Trypanosoma cruzi induced by TcVac4 in a canine model. Santiago HDC, editor. PLoS Negl Trop Dis. 2015;9: e0003625 10.1371/journal.pntd.0003625 25853654 PMC4390229

