# Supplementary material for: Socio-spatial heterogeneity in participation in mass dog rabies vaccination campaigns, Arequipa, Peru
Source: PLoS Negl Trop Dis. 2019 Aug 1;13(8):e0007600. doi: 10.1371/journal.pntd.0007600 (PMC6692050; doi:10.1371/journal.pntd.0007600)
Supplement: S1 Table — (DOCX) [file pntd.0007600.s002.docx]

S1 Table. MDVC communication channels interviewees were exposed by participation level.

| How they learned about the campaign | Vaccinate none dog (n=858) | Vaccinated some dogs (n=170) | Vaccinated all dogs (n=1,397) | p |
| --- | --- | --- | --- | --- |
| Megaphone | 56.9% | 64.5% | 66.5% | <0.001 ^a^ |
| TV | 36.4% | 33.1% | 32.1% | 0.842 ^a^ |
| Radio | 33.9% | 23.3% | 30.4% | 0.122 ^a^ |
| Poster/Banner | 7.8% | 9.9% | 7.4% | 0.337 ^a^ |
| Relative/friend/neighbor | 4.8% | 8.7% | 5.7% | 0.027 ^a^ |
| Newspaper | 1.3% | 0.6% | 1.1% | 0.798 ^a^ |
| Flier | 1.3% | 1.2% | 0.7% | 0.497 ^b^ |
| At municipality | 0.6% | 0.6% | 0.6% | -- |
| Social media | 0.1% | 0.6% | 0.7% | 0.066 ^b^ |
| Community meeting | 0.1% | 0.0% | 0.0% | 0.454 ^b^ |

p-values estimated with ^a^ Chi square test and ^b^ Fisher exact test.
